# The effect of autophagy on the survival and invasive activity of *Eimeria tenella* sporozoites

**DOI:** 10.1038/s41598-019-41947-y

**Published:** 2019-04-09

**Authors:** Nanshan Qi, Shenquan Liao, Asmaa M. I. Abuzeid, Juan Li, Caiyan Wu, Minna Lv, Xuhui Lin, Junjing Hu, Linzeng Yu, Wenwan Xiao, Mingfei Sun, Guoqing Li

**Affiliations:** 1 College of Veterinary Medicine, South China Agricultural University, Guangzhou, 510642 Guangdong, P. R. China; 2 Key Laboratory of Livestock Disease Prevention of Guangdong Province, Scientific Observation and Experiment Station of Veterinary Drugs and Diagnostic Techniques of Guangdong Province, Ministry of Agriculture, Guangzhou, P. R. China; 3 Institute of Animal Health, Guangdong Academy of Agricultural Sciences, Guangzhou, 510640 Guangdong, P. R. China

## Abstract

Autophagy is a cellular process that is vital for the maintenance of homeostasis in eukaryotic cells. Currently, autophagy-related genes (*atgs*) in the *Eimeria tenella* genome database have been reported, but very little is known about the effects of autophagy on the survival and invasive activity of this protozoan. In this study, we investigated the autophagy in *E. tenella* sporozoites under starvation and autophagy-modulators treatments and evaluated the autophagy influence on cellular adenosine triphosphate (ATP) levels, the survival rate and the invasive activity of the sporozoites. The results showed that the autophagy could be induced in the sporozoites by starvation or inducer rapamycin (RP), but it could be inhibited by 3-methyladenine (3-MA) treatment. The sporozoites after starvation and RP-treatment displayed punctate signals of EtATG8 and formed autophagosomes. The survival rate of the sporozoites under starvation was significantly lower than that in the control group, whereas the ATP levels in sporozoite were far greater than those in the control. The quantitative real-time reverse transcriptase polymerase chain reaction (qRT-PCR) showed that the invasive activity of the sporozoites was up- and down-regulated by RP and 3-MA induction, respectively. Our results indicate that autophagy has effects on the survival and invasive activity of *E. tenella* sporozoites, which may provide new insights into anti-coccidial drugs.

## Introduction

Autophagy is a cellular process that is highly conserved among all eukaryotes, allows degradation of defective proteins and organelles, and prevents abnormal proteins accumulation. Autophagy also recycles organelles and proteins (but also lipids) as a nutrient source for the cell during starvation. Moreover, autophagy plays a role in cellular development and differentiation, cell longevity and programmed cell death regulation, degradation of invading pathogens and presenting antigens to the immune system^1^. During autophagy, cytosolic constituents are sequestered in a double-membrane vesicle known as the autophagosome. The autophagosome outer membrane will then fuse with the lysosome to deliver its inner contents, which will be subsequently degraded. Some autophagy-related genes (*atgs*) are only present in specific organism and others represented by orthologues in different eukaryotic cells. Among all ATG proteins, ATG8 is essential for the process of autophagosome formation. This protein exists in a soluble form in the cytoplasm and attaches to the autophagosome membrane upon autophagy induction^2^.

In recent years, some studies on the autophagy of apicomplexan parasites have found that intracellular and extracellular parasites can induce their autophagy to maintain energy balance and cell homeostasis under the starvation condition^3–7^. Moreover, anti-coccidial drugs, such as monensin, can effectively prevent toxoplasmosis by triggering parasite autophagy, which leads to cell dysfunction and programmed cell death^8,9^. However, an anti-malarial drug, chloroquine (CQ), can inhibit the occurrence of autophagy by hindering the fusion of autophagosomes and lysosomes, and consequently mediated the parasite death^10,11^. Therefore, autophagy is very important for the survival of some protozoans. However, there are currently few reports about the effects of autophagy on the survival and invasive activity of *E. tenella*. In this study, we investigated *E. tenella* autophagy marker protein (EtATG8) to explore the effect of autophagy on energy maintenance and invasive activity of *E. tenella* sporozoites to host cell, which may help in developing a new approach to control coccidiosis.

## Results

### The preparation of antibodies against EtATG8

**Figure 1:**
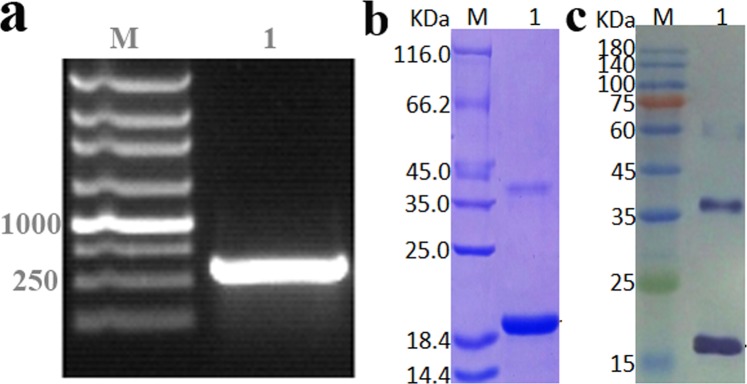
Clone, expression and purification of rEtATG8 protein. (**a**) RT-PCR product. M: DNA marker; 1: *Etatg8*. (**b**) SDS-PAGE analysis of purified protein. M: protein marker; 1: purified rEtATG8. (**c**) Western blot analysis of the rEtATG8 protein, WB was labelled with anti-His-Tag monoclonal antibody. M: prestained marker; 1: rEtATG8.

The ortholog of the *atg8* gene (ETH_00016760) in *E. tenella* genome database was identified as 375-bp (Fig. 1a), which encodes a protein of 124 amino acid residues. The recombinant EtATG8 (rEtATG8), expressed under the induction with 1 mM isopropyl β-D-1- thiogalactopyranoside, was identified as a band of about 18 kDa on 12% SDS-PAGE gel (Fig. 1b), which was consistent with the expected molecular weight. The purified proteins were obtained from the bacterial lysates using a nickel-nitrilotriacetic acid (Ni-NTA) chromatography column. The specific band of about 18 kDa was detected by Western blotting (WB) using the anti-His-Tag monoclonal antibody against His-tagged rATG8 (Fig. 1c). The anti-rEtATG8 polyclonal antibody titer was determined by ELISA after antibody purification. The results showed that the antibody titers reached 1: 51200 after the fourth immunization.

### Starvation-induced autophagy

The newly synthesized ATG8 is present as a soluble form (form I) in the cytoplasm of cells, which forms lipidated proteins (form II) after being biochemically modified by cleavage and lipidation with conjugation to phosphatidylethanolamine (PE). Form I and II of ATG8 can be separated by SDS-PAGE in the presence of urea, and form II (strong hydrophobicity) migrates more rapidly than the form I. Therefore, the form II level can be evaluated by calculating the ratio of form II/form I, and the number of autophagosomes is estimated^5^.

**Figure 2:**
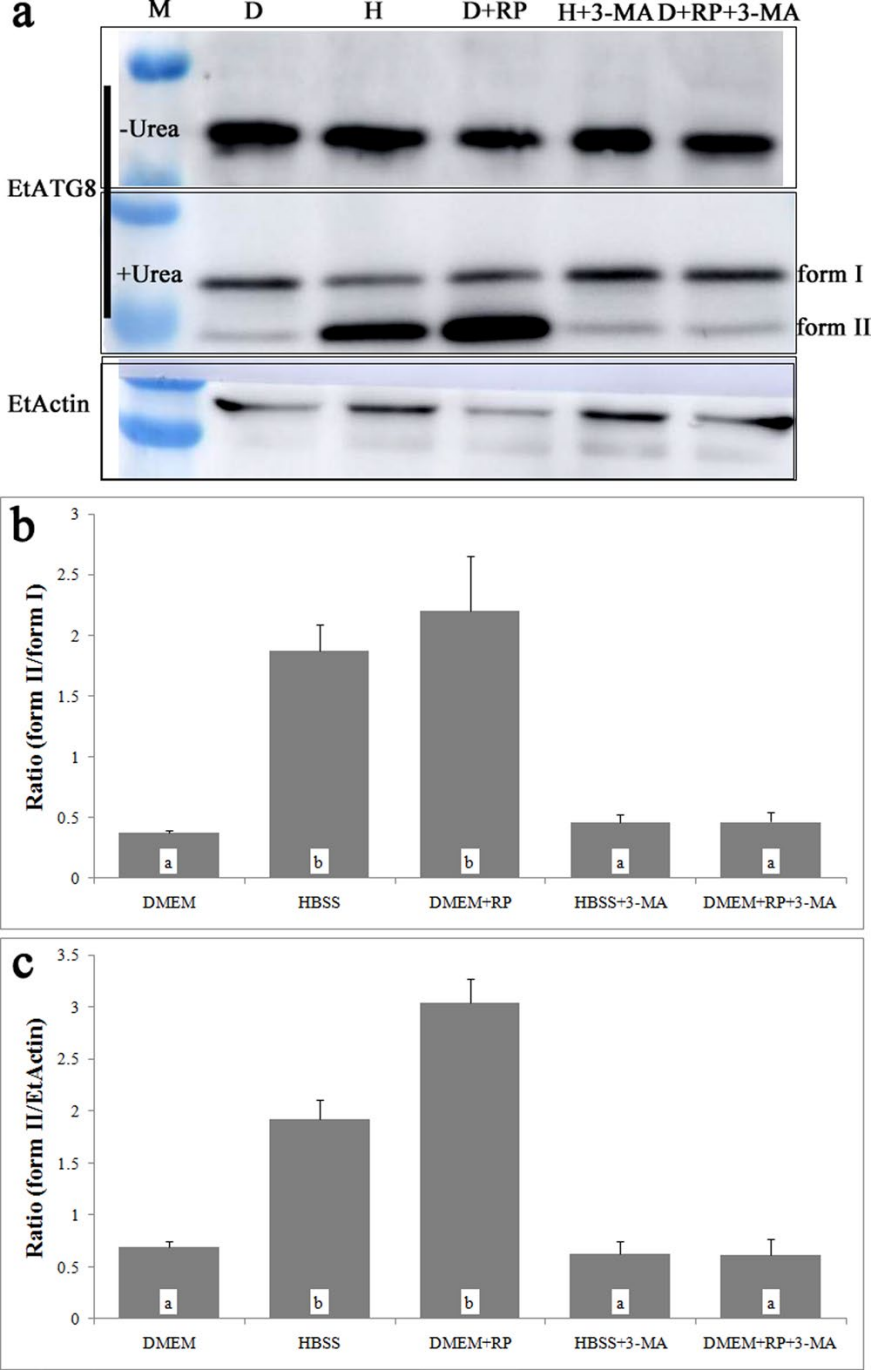
EtATG8 of *E. tenella* sporozoites was lipidated under different conditions. (**a**) The *E. tenella* sporozoites incubated in DMEM, HBSS, DMEM with RP (10 μM), HBSS with 3-MA (25 mM) and DMEM with RP (10 μM) and 3-MA (25 mM) at 41 °C for 8 h. Lysated parasites were fractionated in 12% SDS-PAGE without urea or with 6 M urea. WB was labelled with anti-rEtATG8 antibody or anti-Actin antibody. (**b**) The ratio of EtATG8 form II/form I shown in (a). (**c**) The ratio of EtATG8 form II/ EtActin shown in (a). The ratio of EtATG8 form II/form I or form II/EtActin under different conditions was presented as mean ± SD of three independent experiments, values with different letters are significantly different at *p* < 0.05; values with same letters are insignificantly different at *p* > 0.05 (n = 3).

**Figure 3:**
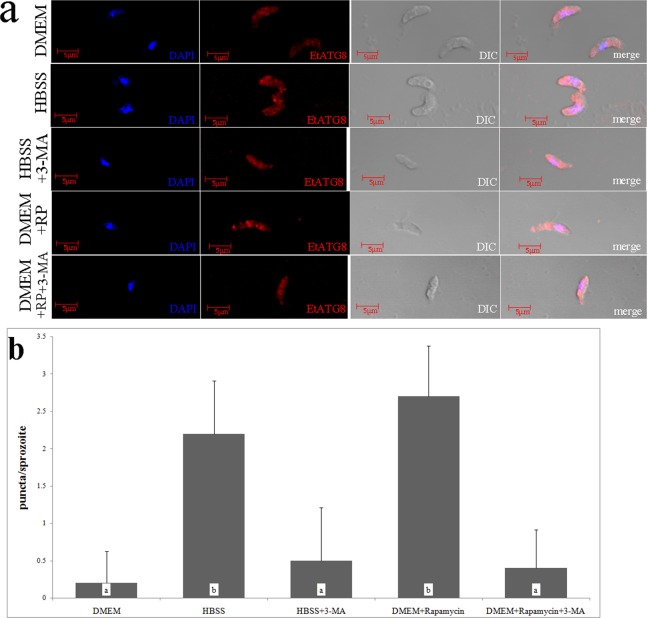
Localization of EtATG8 in *E. tenella* sporozoites. (**a**) Localization of EtATG8 in the sporozoites under different conditions. 1: DMEM; 2: HBSS; 3: HBSS with 3-MA (25 mM); 4: DMEM with RP (10 μM); 5: DMEM with RP (10 μM) and 3-MA (25 mM). (**b**) The average number of autophagosomes in 10 sporozoites was quantified by puncta signals under different conditions and was presented as mean ± SD of three independent experiments, values with different letters are significantly different at p < 0.05; values with same letters are significantly different at p > 0.05(n = 10).

**Figure 4:**
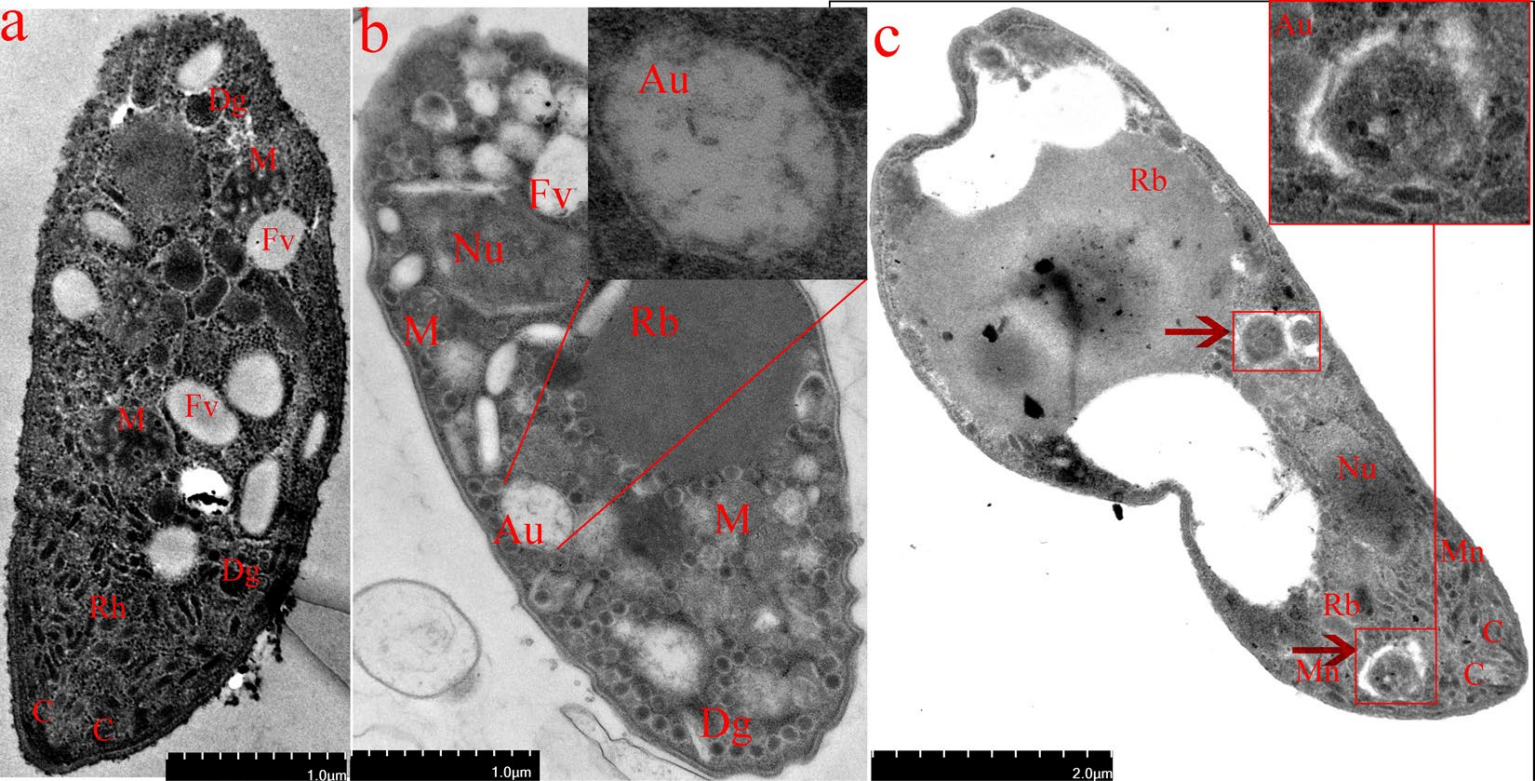
Characterization of autophagosomes under complete DMEM (**a**), HBSS (**b**) and DMEM with RP (**c**) by TEM. C: conoid; Dg: dense granule; Fv: food vacuole; Mn: micronemes; M: mitochondrion; Nu: nucleus; Rb: refraction body; Rh: rhoptry; Au: autophagosome.

The results of subcellular localization of parasite autophagy-related proteins indicated that IFA method can successfully detect the localization of autophagy-related proteins in the parasites and provide evidence for the study of parasite autophagy^12–14^. In this study, we used EtATG8 as an autophagosome marker to investigate the induction of autophagy by starvation in *E. tenella* sporozoites. An amino acid-free isotonic solution (Hank’s balanced salt solution, HBSS) was used for starvation, and the complete Dulbecco’s modified Eagle medium (DMEM) was used as a control. The results demonstrated that EtATG8 was separated into a single band of about 14 kDa by SDS-PAGE without urea under starvation condition (Fig. 2a, upper), whereas it was separated into non-lipidated (form I) and lipidated (form II) proteins by SDS-PAGE with urea (Fig. 2a, middle), and there were no significant increases in form II of ATG8 between the HBSS-induced group with or without CQ (see Supplementary Fig. S1). After starvation for 8 h, the ratio of form II/form I was about 1.87, which was significantly higher than that in DMEM control (0.37) (Fig. 2b), the ratio of form II/EtActin was about 1.93, which was significantly higher than that in DMEM control (0.69) (Fig. 2c), whereas the form II of EtATG8 decreased in the presence of inhibitor 3-MA (Fig. 2a, middle), and the ratio of form II/form I was about 0.47 (Fig. 2b), the ratio of form II/EtActin was about 0.63 (Fig. 2c). The EtATG8 signals were aggregated in the sporozoite cytoplasm after incubation in HBSS, while they were evenly distributed throughout the sporozoites after incubation in DMEM control (Fig. 3a). The average number of starvation-induced autophagosomes (Fig. 3b) was about 2.2, which was significantly higher than that in DMEM control (about 0.2), whereas the average number of induced autophagosomes (about 0.5) decreased in the presence of inhibitor 3-MA (Fig. 3b). After starvation in HBSS, more food vacuoles were formed (Fig. 4b) in sporozoites compared to DMEM control (Fig. 4a), and a double-membrane containing autophagosome-like vesicle (Fig. 4b) appeared in the sporozoites. This finding indicated that starvation can induce autophagosomes formation in *E. tenella* sporozoites.

### Inducer modulated autophagy

To investigate the modulation of autophagy in *E. tenella* sporozoites, we used rapamycin (RP) as an autophagy inducer, 3-methyladenine (3-MA) as an autophagy inhibitor, and the DMEM as a control. On the one hand, WB results presented that form II structures of EtATG8 increased in the presence of inducer RP (Fig. 2a, middle), there were no significant increases in form II of ATG8 between RP-induced group with or without CQ, and the ratio of form II/form I was about 2.2 (Fig. 2b), which was higher than that in DMEM control (0.37), the ratio of form II/EtActin was about 3.0 (Fig. 2c), which was higher than that in DMEM control (0.69). The EtATG8 signals were aggregated in the sporozoite cytoplasm (Fig. 3a). The average number of induced autophagosomes was about 2.7, which was significantly higher than that in the DMEM control (Fig. 3b). The multiple autophagosome-like vesicles (Fig. 4c) appeared in the sporozoites, which consisted of multiple-membrane structures. On the other hand, the form II structures of EtATG8 decreased in the presence of inhibitor 3-MA (Fig. 2a, middle), and the ratio of form II/form I was about 0.47 (Fig. 2b), and the average number of induced autophagosomes (Fig. 3b) was about 0.4. These results demonstrated that the autophagy of *E. tenella* sporozoites can be induced by RP but inhibited by 3-MA.

### Survival of *E. tenella* sporozoites after autophagy induction

**Figure 5:**
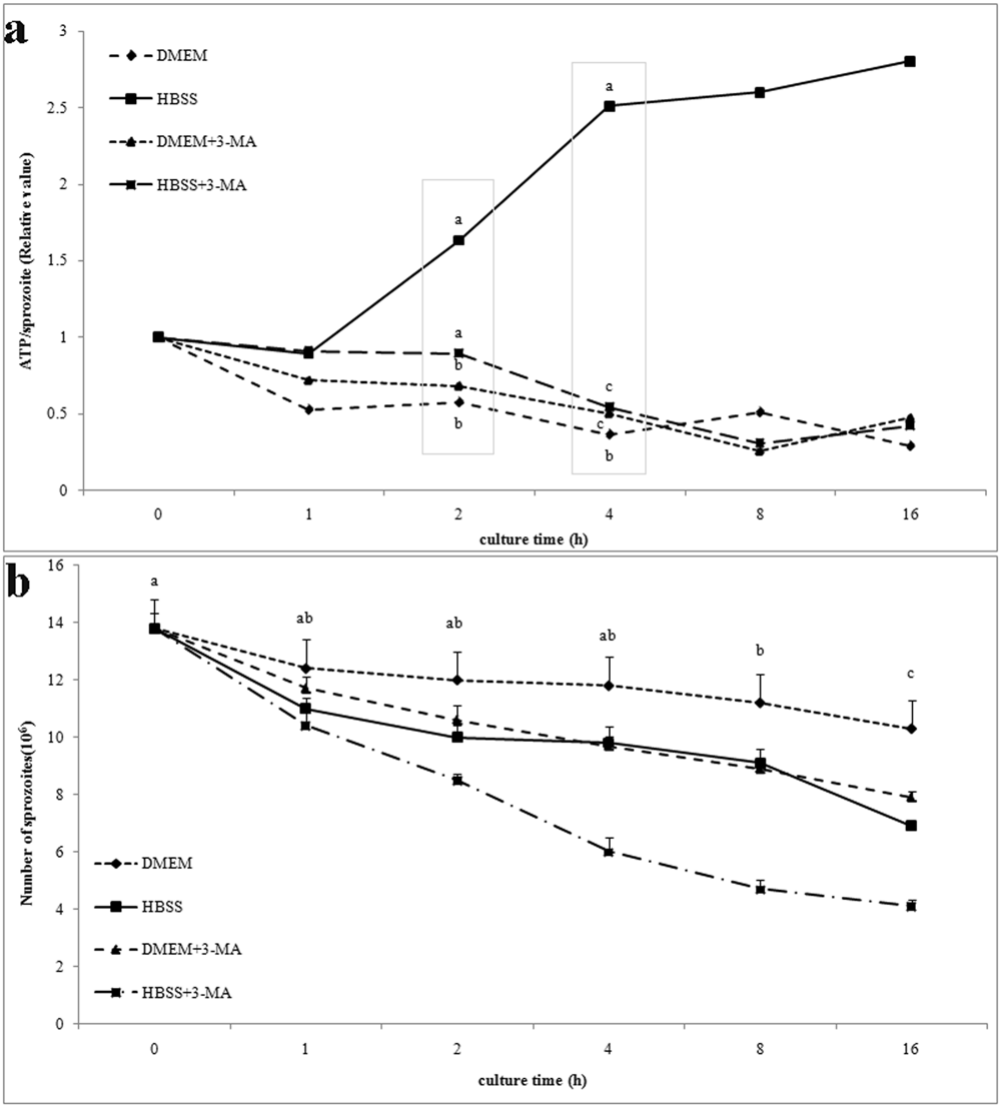
(**a**) The ATP levels in single sporozoites incubated at 41 °C for 1, 2, 4, 8 and 16 h with DMEM, HBSS, DMEM + 3-MA and HBSS + 3-MA, respectively. Values with different letters are significantly different at p < 0.05; values with same letters are insignificantly different at p > 0.05 (n = 4). (**b**) The number of survived *E. tenella* sporozoites after incubation at 41 °C for 0, 1, 2, 4, 8 and 16 h with DMEM, HBSS, DMEM + 3-MA and HBSS + 3-MA, respectively. Values with different letters are significantly different at p < 0.05; values with same letters are significantly different at p > 0.05 (n = 10).

The ATP levels of *E. tenella* sporozoites, treated with starvation medium or autophagy inhibitor, were detected by the chemiluminescence method, but the survival rate of the sporozoites was evaluated by trypan blue staining. Results demonstrated that the ATP level in the sporozoites significantly increased after two-hour starvation and continued to increase in a time-dependent manner, while the ATP level of the sporozoites in the presence of 3-MA decreased significantly (Fig. 5a). After being treated with RP at 10 μM for 8 or 16 hours, the ATP levels of sporozoites were higher than that of the DMEM control group (see Supplementary Fig. S2). The number of survived sporozoites decreased significantly after one-hour starvation and continued to decrease in a time-dependent manner and was furtherly reduced after adding 3-MA (Fig. 5b). The results suggest that starvation- or 3-MA regulated autophagy influenced the survival of *E. tenella* sporozoites.

### Invasive activity of *E. tenella *sporozoite after autophagy induction

**Figure 6:**
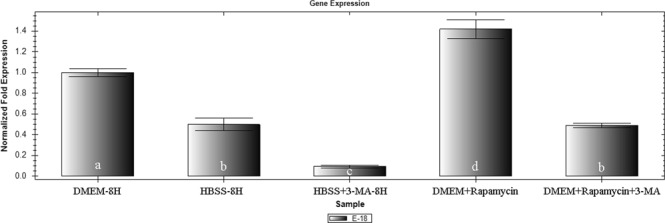
The host cell invasive activity of *E. tenella* sporozoites incubated at 41 °C for 8 h with DMEM, HBSS, DMEM + RP, HBSS + 3-MA and DMEM + RP + 3-MA. Values with different letters are significantly different at p < 0.05; values with same letters are significantly different at p > 0.05 (n = 3).

The *E. tenella* sporozoites were treated with starvation medium or autophagy modulator, the effect of autophagy on the invasive activity of sporozoites were observed using the qRT-PCR method. The results revealed that the sporozoites under the starvation condition in HBSS behaved slightly weaker invasive activity to host cell DF-1 than those in DMEM control, whereas it was reduced significantly in the presence of 3-MA (Fig. 6). The invasive activity of the sporozoites to host cell DF-1 was enhanced significantly after treatment with RP, whereas it was reduced significantly in the presence of 3-MA. The relative expression levels of 18S rRNA gene between sporozoites and host cells were up- and down-regulated by inducer RP and 3-MA, respectively.

## Discussion

Autophagy can both maintain the homeostasis of higher eukaryotic cells under starvation conditions^15,16^ and mediate programmed cell death, so that it plays an important role in cellular activity^17,18^. Recently, studies on autophagy of apicomplexan protozoans, such as *Toxoplasma gondii*, *Plasmodium* spp. and *Trypanosoma* spp., have confirmed the importance of autophagy for parasite survival^5,7,11^. However, very little is known about autophagy in *E. tenella*. The present study found that autophagy of *E. tenella* can be induced by starvation or autophagy inducer RP, whereas the opposite effect was achieved by 3-MA treatment. *E. tenella* sporozoites after starvation or RP-treatment could display punctate signals of EtATG8 and form autophagosomes, whereas 3-MA could block autophagy induced by starvation or RP. These results were consistent with the findings in yeast and higher eukaryotic cells, where the inhibition of the serine/threonine protein kinase TOR (target of RP) can effectively induce autophagy in them^19^. Conversely, the inhibition of phosphoinositide 3-kinase (PI3K) pathway by 3-MA can block autophagosome recruitment^17^.

*E. tenella* is an intracellular parasite whose sporozoites can invade host cells within 24 h after excystation from oocysts^20^ and can get essential nutrients from the host cell. However, the intra-sporocystic sporozoites and extracellular sporozoites search for a new host cell to invade, resulting in a shortage of energy sources^13,21^. In our study on *E. tenlla* sporozoites ATP level after starvation induction, the glucose in HBSS starvation medium was used as a carbon for ATP generation, we detected the ATP level per single sporozoite in each group, which represented the cellular ATP level (ATP/sporozoite). Our results showed that the ATP levels in sporozoites under starvation condition were far higher than those in DMEM control, and the ATP levels of sporozoites after being treated with RP at 10 μM for 8 or 16 hours were higher than that of the DMEM control group (see Supplementary Fig. S2), the similar RP-related effects are also reported by Chin *et al*.^22^. On the contrary, the survival rate of sporozoites under starvation condition at 41 °C for 8 h was significantly lower than that under complete nutrition conditions. Moreover, the starved sporozoites survival rate and ATP levels were significantly reduced in the presence of 3-MA. These results were in agreement with the results reported by Li *et al*.^23^. According to autophagy studies of Ghosh *et al*.^17^ and Yamashita *et al*.^24^, a rapid and irreversible fragmentation of the mitochondrion could be leaded by starvation, and the survival of starvation-encountered sporozoites were reduced because of irreversible damage to mitochondria. In our study, amino acid starvation-induced autophagy mediated increased ATP level, 3-MA-inhibited autophagy mediated decreased ATP level and survival rate of sporozoites, which suggests that sporozoites may maintain the energy balance and further delays their death under starvation conditions by the autophagy mechanism.

Autophagy plays an important role in the parasite invasion, where the organelles (such as mitochondria, microneme, dense granule, and rhoptry) play a key role in providing energy or invasion-related proteins, but some defective organelles recycling is needed during cellular metabolism^3^. Autophagy can maintain the normal cellular homeostasis and cell vitality by the elimination of these defective organelles. In addition, autophagy can degrade the damaged proteins or organelles to produce free amino acids^13,25^, which is conducive for the synthesis of invasion-related proteins. To study the effect of autophagy on the invasive activity of sporozoites, DF-1 cells (2.0 × 10^5^) were infected with the same numbers of sporozoites (4.0 × 10^5^) from each group separately in a 24 -well plate, and then the invasion rate was detected. Our results showed that the sporozoites invasive activity was significantly enhanced in the presence of RP and significantly reduced in the presence of 3-MA. These findings were similar to the results of previous studies^3,4^, where autophagy induced by RP can enhance the invasive activity of apicomplexan sporozoites. While studying the autophagy of *T. gondii*, Lavine *et al*.^8^ found that monensin could inhibit the invasion of *T. gondii* into host cells by inducing autophagy, which was not consistent with our results. The complex mechanism caused this change remains to be studied.

In addition, we found that the invasion rate of sporozoites in the HBSS-treated group was relatively decreased compared to the DMEM control group and significantly decreased with 3-MA treatment than without 3-MA. We hypothesized that the integrity of the mitochondrion is vital for invasion of sporozoites, the invasive activity of starvation-encountered sporozoites can be reduced because of irreversible damage to mitochondria.

In summary, our study confirmed that the autophagy of *E. tenella* sporozoites can be induced by starvation and inducer RP but inhibited by 3-MA and explored the autophagy role in energy maintenance and invasive activity of *E. tenella*. These findings can provide new ideas for developing new anticoccidial drugs.

## Materials and Methods

### Ethics approval

All methods in this study were performed according to the requirements of the Animal Ethics Procedures and Guidelines of the People’s Republic of China and the approval of China Guangdong Province Science and Technology Department (Permit Number: SYXK(Yue) 2011-0116).

### Parasites

The sporulated oocysts of *E. tenella* GD strain were collected and purified as previously described^26,27^. The oocysts were crushed by glass beads, digested by trypsin (0.5%, Sangon Biotech, China) and sodium taurodeoxycholate hydrate (0.5%, Sigma, USA), filtered with a G3 funnel (Jingke, Guangzhou, China) and centrifuged (2152 × g, 10 min) to obtain purified sporozoites. The number of recovered sporozoites was counted three times using a hemocytometer as described previously^28,29^.

### Anti-rEtATG8 antibodies preparation

Total RNA was extracted from *E. tenella* GD strain sporozoites using the HiPure Total RNA Micro kit (MAGEN, China), according to the manufacturer’s instructions. cDNA was transcribed from the extracted RNAs by reverse transcription using PrimeScript II 1st Strand cDNA Synthesis kit (Takara, Japan). The *Etatg8* gene corresponding to the EtATG8 protein was obtained by PCR amplification of *E. tenella* GD strain sporozoites cDNA with the following primers (F: GGCGGATCCATGCCTTCCATAAGAGATGAGAT; R: GGCAAGCTTTTATCCGAGAGTATTTTCTGCG), harboring *Eco*RI and *Xho*I restriction sites, respectively. The PCR products were then cloned into pET28a expression vector (Novagen), and recombinant EtATG8 (rEtATG8) with an N-terminal His tag was expressed in the *Escherichia coli* BL21 strain. The rabbit anti-rEtATG8 serum was prepared as described previously^20,26^. Briefly, four-month- old female rabbits were immunized four times at two weeks interval with rEtATG8 (1.0 mg/rabbit), which was emulsified in complete Freud’s adjuvant for the first immunization. The second, third and fourth immunizations used rEtATG8 (0.5 mg/rabbit) emulsified in incomplete Freud’s adjuvant. Polyclonal rabbit anti-rEtATG8 antibodies were affinity-purified from the rabbit antiserum and then stored at −80 °C until use. The antibody was subsequently used at 1: 500 for WB or IFA.

### Starvation induction and modulation of autophagy

For amino acid starvation-induced autophagy, *E. tenella* sporozoites were incubated in starvation medium, HBSS (137 mM NaCl, 5.4 mM KCl, 0.3 mM Na_2_HPO_4_, 0.4 mM KH_2_PO_4_, 1.3 mM CaCl_2_, 0.6 mM MgSO_4_, 4.2 mM NaHCO_3_, 5.6 mM glucose, pH 7.4), at 41 °C for 0, 1, 2, 4, 8 and 16 h as test groups. The sporozoites were incubated in DMEM at 41 °C for the same time periods and were used as a control. For modulation of autophagy, RP (Sigma, USA) was used as an autophagy inducer, and 3-MA was used as an autophagy inhibitor. The *E. tenella* sporozoites were incubated in DMEM with rapamycin at 10 μM (DMEM + RP), DMEM with 3-MA at 25 mM (DMEM + 3-MA), HBSS with 3-MA at 25 mM (HBSS + 3-MA), and DMEM with RP at 10 μM and 3-MA at 25 mM (DMEM + RP + 3-MA) separately at 41 °C for 8 h and used as test groups, while sporozoites incubated in DMEM or HBSS at 41 °C for the same time period were used as a control.

### Immunoblot analysis

Following the induction of starvation and/or inducer treatment, the total proteins from sporozoites incubated in DMEM, HBSS, DMEM + RP, HBSS + 3-MA, and DMEM + RP + 3-MA were prepared using the Cell Lysis Buffer system (Beyotime, China). The protein concentrations were determined using the BCA protein assay kit (Beyotime, China). After that, total protein extracts of each sample (100 μg) were separated on 12% SDS-PAGE with 6 M urea to visualize the lipid-conjugated form of EtATG8, or on 12% SDS-PAGE without urea to visualize the total EtATG8. The EtATG8 was determined by WB with polyclonal rabbit anti-rEtATG8 antibody (diluted 1:500). A rabbit monoclonal anti-Actin antibody (diluted 1:500, Sigma, USA) was used as the control, the goat anti-rabbit IgG-HRP (diluted 1:2000, Santa Cruz Biotechnology, USA) was used as the secondary antibody. The expressions of EtActin, EtATG8 form I and II were quantified by densitometry, and the relative expression of EtATG8 form II was determined as the ratio of form II/I and form II/EtActin. All assays were performed in triplicate.

### Immunofluorescence analysis (IFA)

IFAs were performed on the sporozoites incubated in DMEM, HBSS, DMEM + RP, DMEM + 3-MA, HBSS + 3-MA, and DMEM + RP + 3-MA as described previously^7,14,25^. In brief, the extracellular sporozoites were harvested after being incubated under the above-mentioned conditions. The parasites were fixed with 4% (w/v) paraformaldehyde for 20 min in phosphate-buffered saline (PBS), permeabilized with 1% Triton X-100 for 10 min after adherence to poly-L-lysine slides for 20 min and then blocked with 0.1% (w/v) BSA in PBS for 30 min at 37 °C. The parasites were then incubated with the anti-rEtATG8 antibody (diluted 1:500) and donkey anti-rabbit IgG-R (diluted 1:400, Santa Cruz Biotechnology, USA) for 1 h at 37 °C. The parasite DNAs were labeled with 10 μg/ml of the fluorescent DAPI (4′,6-diamidino-2-phenylindole) solution (Beyotime, China) for 5 min, and all images were acquired with the Zeiss LSM710 confocal system (CarlZeiss). The average number of autophagosomes in at least ten sporozoites was quantified by punctate EtATG8 signals per sporozoite in each experimental set, and three independent experiments were performed.

### Transmission electron microscope (TEM)

Following incubation in DMEM, HBSS or DMEM + RP (10 μM) at 41 °C for 8 h, *E. tenella* sporozoites were fixed in 2.5% glutaraldehyde and 4% paraformaldehyde in 0.1-M cacodylate buffer for 3 h, and post-fixed in 1% osmium tetroxide in the same buffer for 3 h. Parasites were dehydrated in graded series of ethanol and then treated with 100% epoxyethane, infiltrated with Spurr’s resin and polymerized for 12 h at 70 °C. Ultra-thin sections (70 nm) were prepared and stained with 1% uranylacetate and 0.25% lead citrate. Finally, these sections were visualized under a Hitachi HT7700 transmission electron microscope (TEM, HITACHI, Japan).

### Detection of the ATP level and survival of sporozoite

Following the induction of starvation and/or inducer treatment, an ATP Assay Kit (Beyotime, China) was used to detect the ATP level of the sporozoites incubated in DMEM, HBSS, DMEM + 3-MA and HBSS + 3-MA at 41 °C for 1, 2, 4, 8 and 16 h by the chemiluminescence method. The live sporozoites under different conditions were determined by trypan blue exclusion^29,30^. Shortly, 0.02 ml sporozoite suspensions (2.77 × 10^6^ sporozoites/ml) were mixed with equal volumes of 0.08% trypan blue solution and loaded into a hemocytometer for immediate examination, and three independent experiments were performed.

### Impact of autophagy on the invasive activity of sporozoites

The chicken embryo fibroblast cell line DF-1 (ATCC) was used for the host cell invasion assay to analyze the autophagy effect on *E. tenella* sporozoite invasive activity. Sporozoites were incubated at 41 °C for 8 h with different treatments (DMEM, HBSS, DMEM + RP, HBSS + 3-MA and DMEM + RP + 3-MA). DF-1 cells (2.0 × 10^5^) were infected with sporozoites (4.0 × 10^5^) in a 24 -well plate (Corning, USA). The cells were washed with 0.01 M phosphate-buffered saline (PBS) and collected after cultivation at 41 °C for 10 h. The invasion efficiency in each of the above-mentioned treatments was evaluated using qRT-PCR with SYBR green I detection as described previously^31^. Concisely, the primers (Table 1) were designed using Primer Premier 5.0, and qRT-PCR was performed on the CFX Connect system (Bio-Rad, USA). The *Et*18*S rRNA* gene was used as the detection gene. Double-distilled H_2_O (ddH_2_O) and chicken *18* *S rRNA*gene were used as the negative and internal controls, respectively. All assays were carried out in triplicate. Finally, the invasive activity was assessed by the relative expression levels of *18S rRNA* genes between the sporozoites and host cells^31^.

**Table 1:** Primer sequences used in this study.

Gene ID	Forward primer (5′–3′)	Reverse primer (5′–3′)
qRT-PCR (*chicken18S rRNA*)	TGTGCCGCTAGAGGTGAAATT	TGGCAAATGCTTTCGCTTT
qRT-PCR (*Et18S rRNA*)	CTGAGAAACGGCTACCACATC	TGACCACGACAGAAATCCAAC

### Statistical analyses

For the quantification of the Western blot bands, autophagosome number, the ATP levels, the survival rate, and the invasive activity of *E. tenella* sporozoites, three independent experiments were performed, and the data were statistically analyzed with one-way ANOVA, values with different letters were significantly different (p < 0.05); values with same letters were insignificantly different (p > 0.05).

## Supplementary information

Supplementary Information

## Data Availability

All relevant data were included in the paper.
