# Supplementary material for: The effect of autophagy on the survival and invasive activity of Eimeria tenella sporozoites
Source: Sci Rep. 2019 Apr 9;9:5835. doi: 10.1038/s41598-019-41947-y (PMC6456608; doi:10.1038/s41598-019-41947-y)
Supplement: Supplementary file 1 — Supplementary Information [file 41598_2019_41947_MOESM1_ESM.docx]

**The effect of autophagy** **on the survival and invasive activity of *Eimeria tenella* sporozoites**

Nanshan Qi^1, 2^, Shenquan Liao^2^, Asmaa M.I.Abuzeid^1^, Juan Li^2^, Caiyan Wu^2^, Minna Lv^2^, Xuhui Lin^2^, Junjing Hu^2^, Linzeng Yu^2^, Wenwan Xiao^2^, Mingfei Sun^2*^, Guoqing Li**^1*^**

^1^College of Veterinary Medicine, South China Agricultural University, Guangzhou 510642, Guangdong, P R China

^2^Key Laboratory of Livestock Disease Prevention of Guangdong Province,

Scientific Observation and Experiment Station of Veterinary Drugs and Diagnostic Techniques of Guangdong Province, Ministry of Agriculture, P. R. China

Institute of Animal Health, Guangdong Academy of Agricultural Sciences, Guangzhou 510640, Guangdong, P. R. China

Email addresses of all authors: [nanshanqi@163.com](mailto:nanshanqi@163.com) (Nanshan Qi), [454092624@qq.com](mailto:454092624@qq.com) (Shenquan Liao), asmaa_ibrahim@vet.suez.edu.eg (Asmaa M.I.Abuzeid), [153047901@qq.com](mailto:153047901@qq.com) (Juan Li), [396386408@qq.com](mailto:396386408@qq.com) (Caiyan Wu), [673767026@qq.com](mailto:673767026@qq.com) (Minna Lv), [96229015@qq.com](mailto:96229015@qq.com) (Xuhui Lin), [1417430871@qq.com](mailto:1417430871@qq.com) (Junjing Hu), [546026100@qq.com](mailto:546026100@qq.com) (Linzeng Yu) and [942242920@qq.com](mailto:942242920@qq.com) (Wenwan Xiao).

*Authors for correspondence: [gqli@scau.edu.cn](mailto:gqli@scau.edu.cn) (Guoqing Li) and smfei7810@126.com (Mingfei Sun).

**Supplementary Information**

**Supplementary Figure legends**

**Supplementary Figure S1.** EtATG8 of *E. tenella* sporozoites was lipidated under different conditions: incubated in DMEM (D), HBSS (H), DMEM with Rapmycin (D+RP) at 10μM, HBSS and Chloroquine (H+CQ), DMEM with Rapmycin and CQ (D+RP+CQ), and DMEM with CQ (D+CQ) at 41°C for 8 h. Lysated parasites were fractionated in 12% SDS-PAGE with 6 M urea. WB was labelled with anti-rEtATG8 antibody or anti-Actin antibody.

**Supplementary Figure S2.** The ATP levels in single sporozoite incubated in HBSS and RP (10μM), at 41°C for8 or 16 h, respectively, values with different letters are significantly different at *p*<0.05; values with same letters are in significantly different at *p*>0.05 (n=3).

**Supplementary Figure S1**


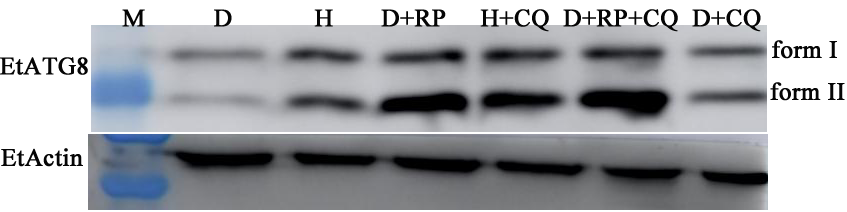


**Supplementary Figure S2**

**
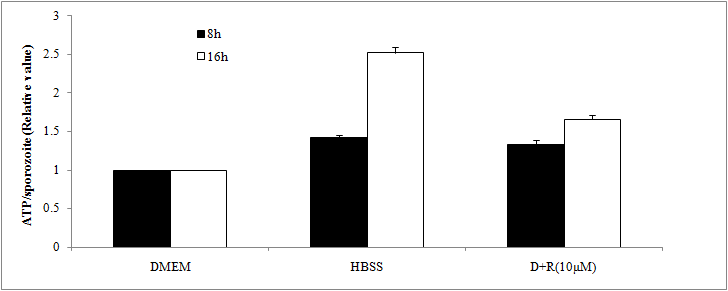
**
